# Sulodexide recovers endothelial function through reconstructing glycocalyx in the balloon-injury rat carotid artery model

**DOI:** 10.18632/oncotarget.20518

**Published:** 2017-08-24

**Authors:** Tianjia Li, Xinnong Liu, Zhewei Zhao, Leng Ni, Changwei Liu

**Affiliations:** ^1^ Department of Vascular Surgery, Peking Union Medical College Hospital, Chinese Academy of Medical Sciences and Peking Union Medical College, Beijing 100005, China

**Keywords:** glycocalyx, sulodexide, endothelial repair, balloon injury, inflammation

## Abstract

Disruption of endothelial cell function is a principle event in cardiovascular disease. Accordingly, therapies have mostly focused on repairing the endothelium, but little attention has been paid to the reconstruction of glycocalyx, which covers the endothelium and protects the function of endothelial cells. Sulodexide has a similar glycosaminoglycan structure to glycocalyx, so it is assumed to be effective in remodeling the glycocalyx following damage. We assessed the effect of sulodexide on glycocalyx remodeling and endothelial function in the balloon-injury rat carotid artery model. Electron micrographs showed that sulodexide (2mg/kg, administered by intraperitoneal injection for seven days after injury) could reconstruct the endothelial glycocalyx and recover the clear cytoarchitecture. With regard to endothelial function, sulodexide increased endothelial nitric oxide synthase level, attenuated endothelial hyperplasia, and inhibited platelet aggregation that benefitted from glycocalyx reforming. Sulodexide decreased the glycocalyx damage related expression of CD31 and intercellular cell adhesion molecule-1 in endothelium, accompanying by the downregulation of leukocyte counts and C-reactive protein levels. The levels of the atherosclerosis-related factors, osteopontin and vascular cell adhesion molecule-1, which increased in activated endothelial cells lacking glycocalyx, were normalized by sulodexide. Along with the benefit of glycocalyx reconstruction, sulodexide reversed the dyslipidemia. Moreover, sulodexide prevented CD68-positive inflammatory cells infiltration into the vascular wall, presumably as a result of glycocalyx reconstruction. In summary, sulodexide treatment reconstructed glycocalyx which therefore preserved endothelial function and attenuated the expression of inflammatory factors, and decreased the blood coagulation and lipid metabolism, all of which are important for vascular healing.

## INTRODUCTION

**Figure 1:**
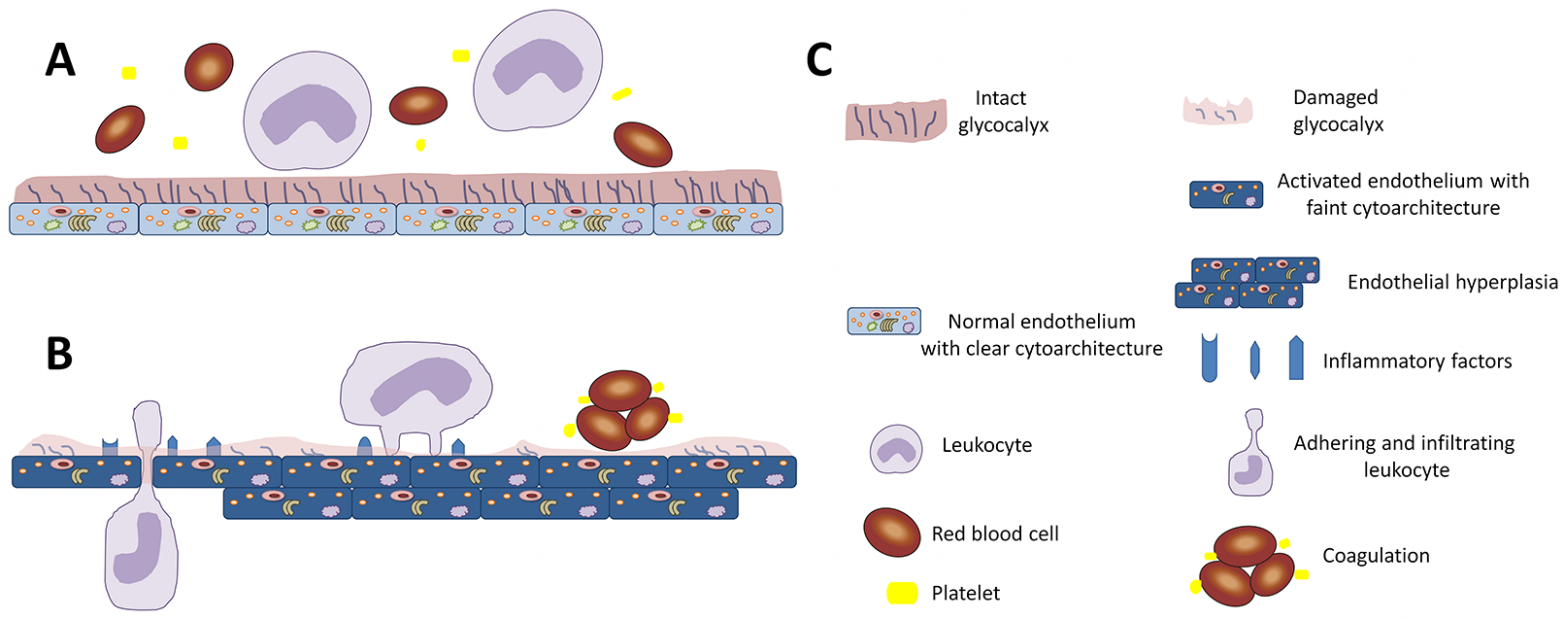
Diagrammatic representation of the importance of glycocalyx in endothelial function **(A)** The intact glycocalyx shields and isolates endothelial cells from circulating cells, and maintains the normal function of the endothelium. **(B)** Damage to the glycocalyx exposes the underlying endothelial cells resulting in hyperplasia. In addition, the relatively activated endothelial cells express inflammatory factors, induce the adhesion and infiltration of leukocytes, and cause coagulation. **(C)** Key for the schematic.

Endothelial cells perform key homeostatic functions such as regulating blood flow, lipid metabolism, and interaction with inflammatory cells. Previous studies have mostly focused on the endothelium, but it has been shown that the neointima can sometimes be compromised. The primary interface between the blood and the endothelium is formed by an endothelial surface layer, referred to as the glycocalyx. The glycocalyx is a network of membrane-bound proteoglycans, covering the luminal side of the endothelium. It forms a negatively charged gel-like surface structure of proteoglycans with covalently bound polysaccharide chains, referred to as glycosaminoglycans, glycoproteins, and glycolipids. Its main carbohydrate constituents are heparan sulfate, chondroitin sulfate, and hyaluronic acid. Glycocalyx has proven to be important for normal physiological function of the endothelium and therefore governs the physiological function of blood vessels [1]. As shown in Figure 1, glycocalyx acts as an interactive scaffold that regulates the interaction with circulating blood cells and thereby serves as a key modifier of endothelial function. This carbohydrate-rich gel-like structure affects the expression of inflammatory factors and regulates the function of the endothelium. Proteins that are affected by glycocalyx integrity are involved in cell attachment, migration, inflammation, blood coagulation, and lipid metabolism, thus putting the glycocalyx at the center of the cardiovascular pathophysiology.

While endothelial glycocalyx serves a normal physiological adaptive function, glycocalyx maladaptation has been identified as an important mechanism in the progression of cardiovascular diseases. Being a protective layer between endothelial cells and blood, damage to the glycocalyx during vascular surgery has been postulated to cause endothelial hyperplasia, thrombus, stenosis, and arteriosclerosis. Previous studies have shown that, the level of syndecan-1, a marker of glycocalyx degradation, is associated with increased mortality in trauma patients [2], and damage to the glycocalyx occurs in ischemia-reperfusion of the coronary artery [3]. Therefore, events that occur following damage to the glycocalyx are being studied to prospectively identify the risk of cardiovascular events, and furthermore the effect of rebuilding the glycocalyx on the prevention and treatment of cardiovascular diseases is also being actively studied.

During synthesis of the glycocalyx, both endothelium- and plasma- derived molecules integrate into the mesh. According to some studies, exogenous administration of glycocalyx constituents, such as chondroitin sulfate and hyaluronic acid, can help reconstruct an impaired glycocalyx [4]. In this regard, sulodexide is a highly purified mixture of glycosaminoglycans, which is comprised of dermatan sulfate and low molecular weight heparin. Sulodexide can bind to endothelial cells and has anti-thrombotic and pro-fibrinolytic activities [5, 6]. Because its structure is similar to the constituent component in glycocalyx, it can be considered as being able to provide precursors for glycocalyx repair. In addition, recent studies have demonstrated that sulodexide may also exert favorable effects on restoring endothelial function, has an anti-inflammatory action, and can regulate the expression of cytokines and chemokines [7, 8]. An attempt has been made in type 2 diabetes mellitus to reverse glycocalyx dysfunction by administering sulodexide [9]. In this study, the transcapillary rate of albumin escape was reduced in the sulodexide-treated group. Although the reversal of glycocalyx abnormalities in diabetes is only partial, this approach also holds promise and invites further work.

As noted above, it is reasonable to propose that administration of sulodexide will play a protective role in reconstructing damaged glycocalyx. The aim of this study was to examine the protective effort of sulodexide on glycocalyx reconstruction and on resulting endothelial function. Because structural aspects as well as biochemical composition determine the properties of glycocalyx, *in vivo* experiments are required to explore its physiological function. Apart from this, because of its dynamic and fragile structure, glycocalyx is unstable after being removed from the *in vivo* setting. One further complication is that glycocalyx can be easily lost during dehydration, sectioning, and staining procedures. Therefore, optimal preservation and visualization of glycocalyx requires the perfusing fixation, which is not possible with patient biopsy material, but is feasible in animal studies. Here, we used the balloon-induced rat carotid artery injury model to mimic vascular surgery, in which the endothelium and glycocalyx were all shed. We examined reconstruction of the glycocalyx using electron microscopy. We then evaluated endothelial healing, endothelial function, and the expression of inflammatory factors. In addition, blood samples were collected to assess coagulation status, inflammatory status, and lipid metabolism.

## RESULTS

### Sulodexide promotes reconstruction of the glycocalyx

**Figure 2:**
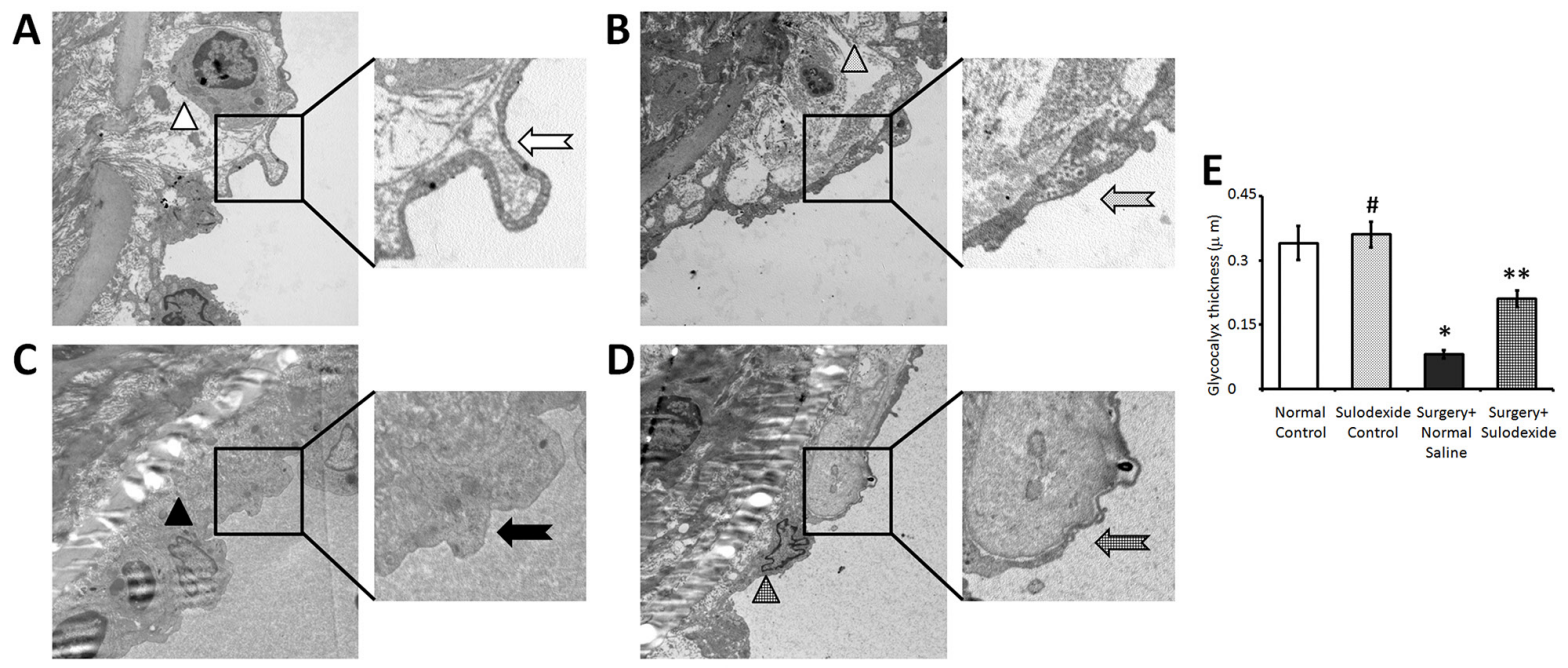
Sulodexide reconstructs the endothelial glycocalyx shown by electron microscopy All images are the luminal side of endothelium. **(A)** The control group with normal feeding and without surgery had a clear cellular structure (white triangle) that was coated with glycocalyx (white arrow). **(B)** The sulodexide control group, that received intraperitoneal injections of sulodexide without surgery, also had a clear cellular structure (spotted triangle) that was coated with glycocalyx (spotted arrow). **(C)** The surgery+normal saline group, that had a balloon-induced injury and were injected with normal saline for seven days, showed faint cytoarchitecture and indistinct organelles (black triangle), and a bare endothelium lacking glycocalyx (black arrow). **(D)** The surgery+sulodexide group, that had a balloon-induced injury and were injected with sulodexide for seven days, had an intact cytoarchitecture (grid triangle) and a partially reconstructed glycocalyx (grid arrow). **(E)** Quantitative analysis of the glycocalyx thickness. 10000 × magnification. #P>0.05 sulodexide control versus normal control, *P<0.05 surgery+normal saline versus normal control, **P<0.05 surgery+sulodexide versus surgery+ normal saline.

As can be seen from the electron microscopy images, the normal carotid intima is coated with an integrated glycocalyx (Figure 2A and 2B), which is continuous and uniform. After balloon-injury, although part of the endothelial layer had recovered in the surgery+ normal saline group, there were still bare areas on the endothelial cells indicating a lack of glycocalyx (Figure 2C). Moreover, the endothelial cells that lacked glycocalyx had poor cytoarchitecture and indistinct organelles, indicative of reduced endothelial activity and function. Remarkably, in the sulodexide+surgery group (Figure 2D), there was a significant glycocalyx reconstruction, consistent with an improvement in organelle appearance, over the same recovery time following balloon-injury. These data are consistent with the role of the glycocalyx in maintaining the normal function of endothelial cells.

### Sulodexide facilitates the functional and constructive recovery of endothelium through reconstructing glycocalyx

**Figure 3:**
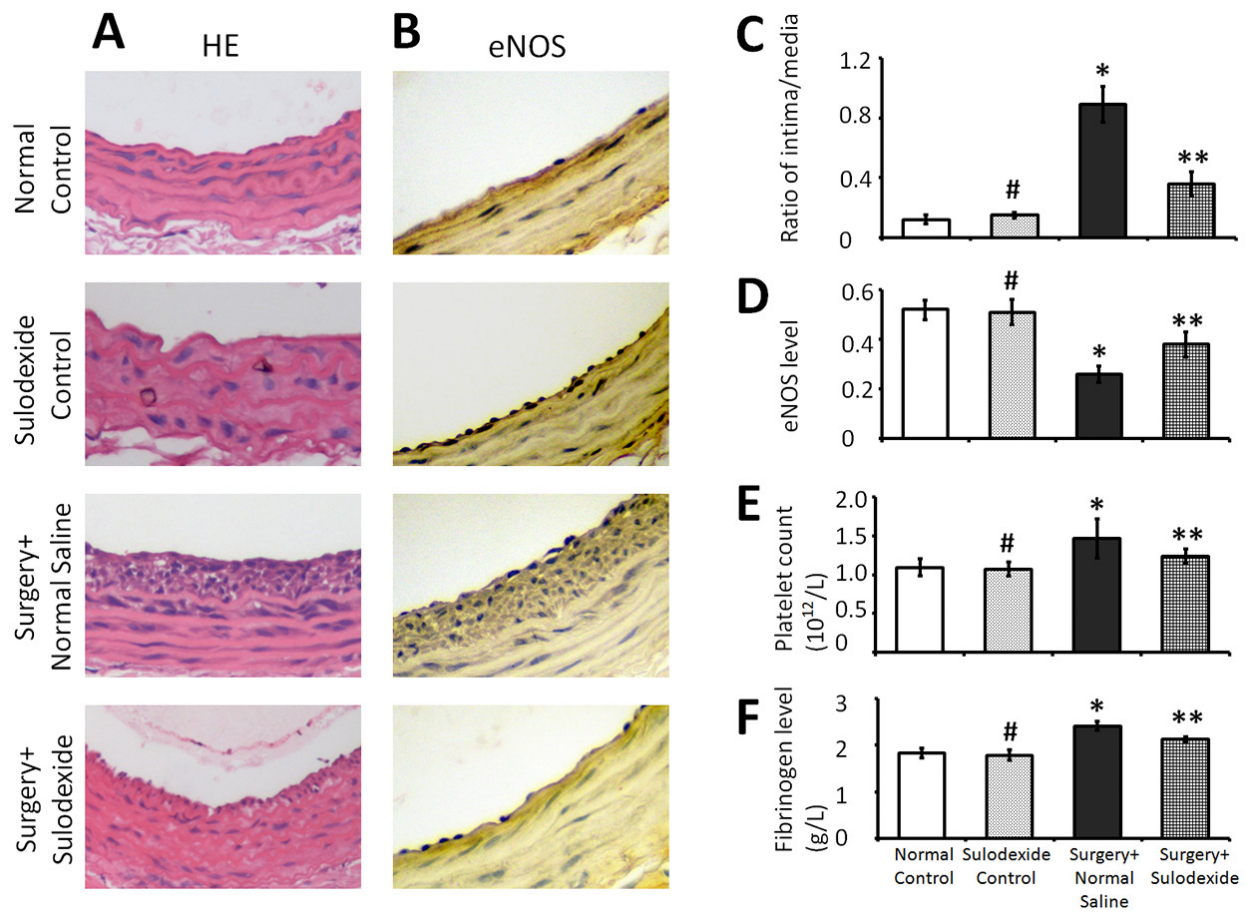
Sulodexide promotes the constructive and functional recovery of balloon-injury endothelium, and regulates coagulation The internal carotid arteries samples from rats were taken for pathological examination and quantitative analysis. Blood samples were also collected to measure the levels of coagulation related parameters. **(A)** Representative HE (400 × magnification) staining which was used to detect the endothelial hyperplasia. **(B)** Representative IHC (400 × magnification) staining for eNOS levels which was used to analyze functional recovery of the endothelium. **(C)** Quantitative analysis for the intima/media ratio from images such as those shown in A. **(D)** Quantitative analysis of the IOD values representing eNOS levels from images such as those shown in B. **(E)** Serum platelet levels. **(F)** Serum fibrinogen levels. #P>0.05 sulodexide control versus normal control, *P<0.05 surgery+normal saline versus normal control, **P<0.05 surgery+sulodexide versus surgery+ normal saline.

The recovery of the endothelium includes both an integrated construction process and a return to normal function. The integrity of the endothelial layer not only relies on the timely recovery of the endothelium, but is also dependent on restrained hyperplasia, which can cause vascular stenosis and dysfunction. In the Figure 3A, the surgery+normal saline group showed evidence of neointimal hyperplasia compared with the normal control group, whereas the surgery+sulodexide group regained a normal endothelial structure. Normal endothelial cell function relies on the activity of endothelial nitric oxide synthase (eNOS), which produces nitric oxide (NO) and is responsible for regulating the physiological function of the vascular wall. The surgery+normal saline group had evidence of excessive endothelial cell proliferation, but had clearly lower levels of eNOS compared to the control groups (Figure 3B and 3D). Conversely, in the surgery+sulodexide group, the balloon-injury damaged carotid artery recovered its natural structure and the levels of functional eNOS returned to normal (Figure 3B and 3D).

An important role for endothelial cells in blood vessels is the inhibition of thrombosis. With regard to the levels of coagulation factors, the amount of platelets in the surgery+normal saline group was higher than in the control group, whereas this number decreased in the surgery+sulodexide group (P=0.02, P<0.05, Figure 3E). The coagulation function of platelets relies on fibrinogen, which is a key factor in the formation of blood clots. Balloon-injury surgery increased the blood fibrinogen levels compared with the control group (P=0.03, P<0.05, Figure 3F), but sulodexide down-regulated these levels (P=0.02, P<0.05, surgery+sulodexide versus surgery+normal saline). The elevated levels of these pro-coagulation factors are a response to the injury-related inflammation. In this regard, sulodexide would be able to attenuate the occurrence of vascular disease by aiding in the reconstruction of the glycocalyx.

### Sulodexide attenuates the balloon-injury related inflammation in endothelium through reconstructing glycocalyx

**Figure 4:**
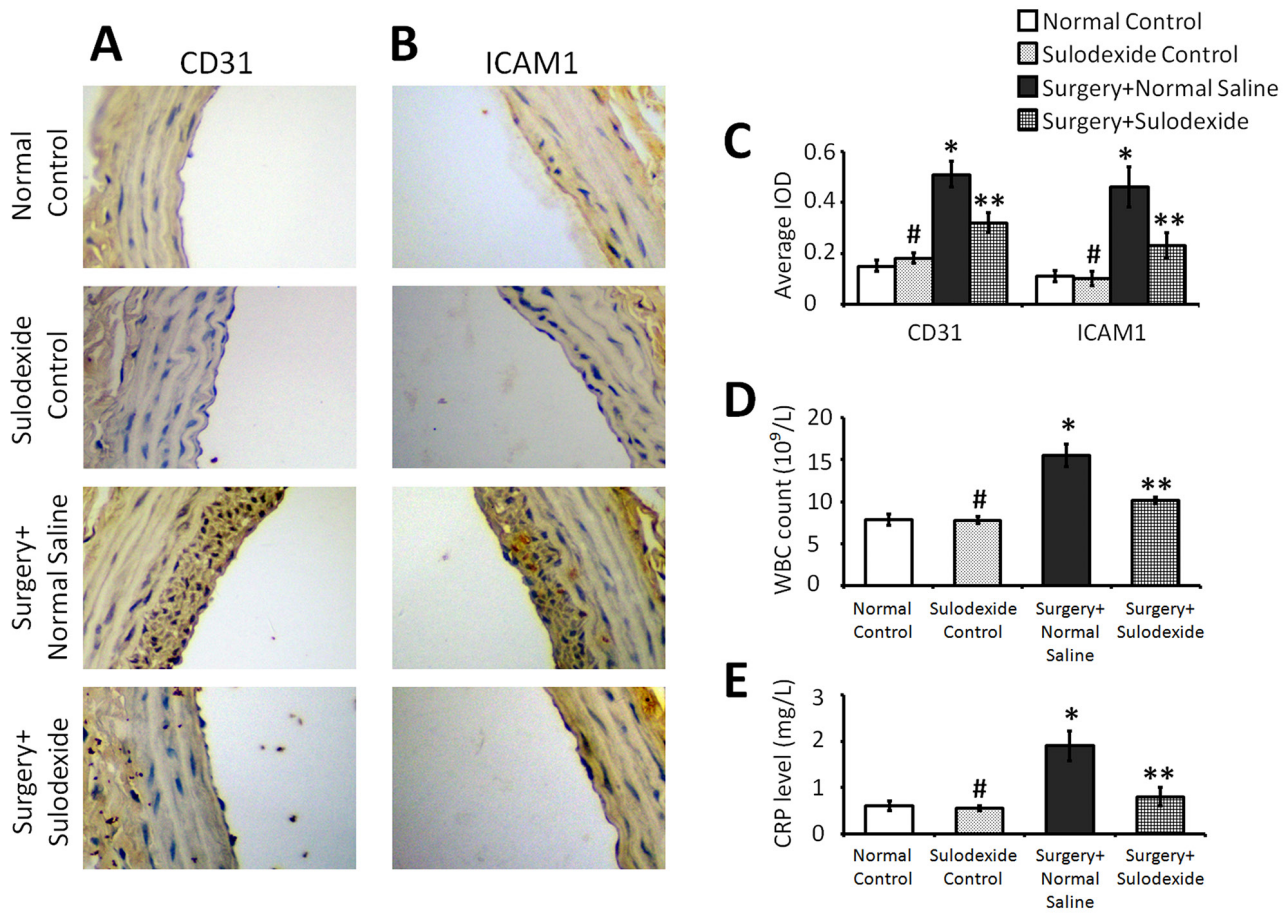
Sulodexide attenuates the balloon surgery-induced expression of the inflammatory factors CD31 and ICAM1, and provides a systemic anti-inflammatory effect **(A)** Representative IHC (400 × magnification) staining for CD31. **(B)** Representative IHC (400 × magnification) staining for ICAM1. **(C)** The average IOD values for CD31 and ICAM1. **(D)** Serum WBC count. **(E)** Serum CRP level. #P>0.05 sulodexide control versus normal control, *P<0.05 surgery+normal saline versus normal control, **P<0.05 surgery+sulodexide versus surgery+ normal saline.

The balloon-injury affects the endothelial function which is accompanied by the expression of inflammatory factors. CD31 is part of the intercellular junction in the inflammation-activated endothelium, and is involved in leukocyte adhesion and migration [10, 11]. As shown in Figure 4A, the expression of endothelial cell CD31 was significantly increased after balloon injury in the surgery+normal saline group, whereas sulodexide significantly attenuated this injury-induced expression (P=0.001, P<0.05, surgery+sulodexide group versus surgery+normal saline group). The intercellular cell adhesion molecule-1 (ICAM1) is a cell surface glycoprotein which typically mediates the interaction between endothelial cells and leukocytes. The levels of ICAM1, similar to CD31, were significantly lowered in the surgery+sulodexide group compared to the surgery+normal saline group (P=0.001, P<0.05, Figure 4B and 4C). For the surgery-related inflammation, both white blood cell (WBC) counts and the level of C-reactive protein (CRP) were elevated in surgery+normal saline group, while were alleviated in surgery+sulodexide group (P=0.01, P<0.05, Figure 4D and 4E). As the importence of glycocalyx for endothelium, the down-regulation of these inflammatory responses is attribute to the effect of sulodexide in glycocalyx reconstructing.

### Sulodexide attenuates the atherosclerotic factors and regulates lipid metabolism

**Figure 5:**
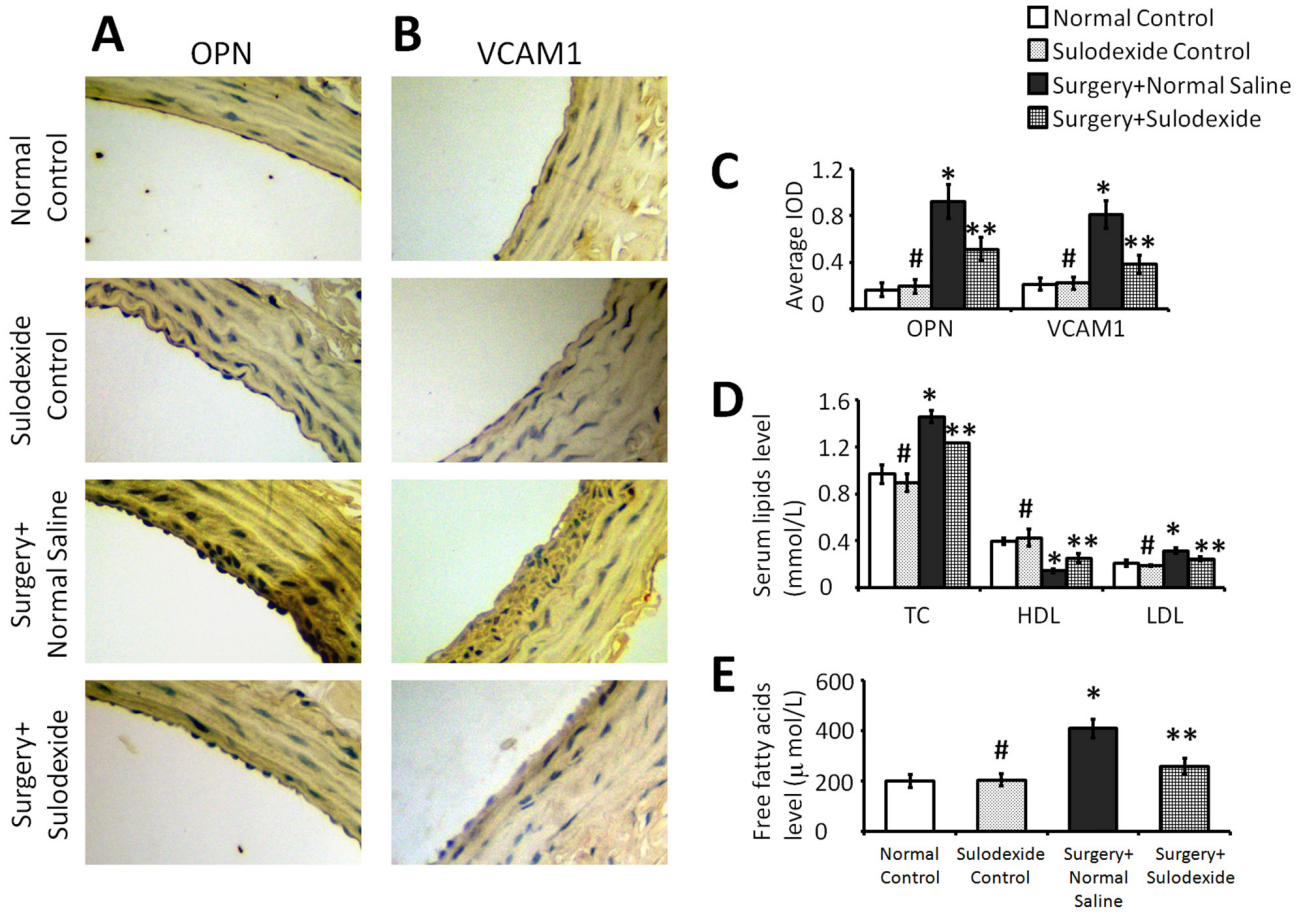
Sulodexide inhibits the atherosclerosis-related factors OPN and VCAM1, and regulates lipid metabolism **(A)** Representative IHC (400 × magnification) staining for OPN. **(B)** Representative IHC (400 × magnification) staining for VCAM1. **(C)** The average IOD values of OPN and VCAM1. **(D)** Serum lipids TC, LDL, and HDL. **(E)** Serum free fatty acids. #P>0.05 sulodexide control versus normal control, *P<0.05 surgery+normal saline versus normal control, **P<0.05 surgery+sulodexide versus surgery+ normal saline.

The intact endothelial glycocalyx can not only inhibit thrombosis and inflammation, but can also protect the blood vessel from the attachment and deposition of lipids, which can cause atherosclerosis and pathologic calcification. Vascular cell adhesion molecule 1 (VCAM-1) is a cell surface sialoglycoprotein that is expressed by the activated endothelium. This membrane protein mediates leukocyte-endothelium adhesion and signal transduction, and plays a role in the development of atherosclerosis. Osteopontin (OPN) is a matricellular protein that is closely related to calcium metabolism and pathologic calcification [12, 13]. As shown in Figure 5, the expression of VCAM1 and OPN were significantly increased in the surgery+normal saline group, and sulodexide was able to significantly attenuate the expression of these two atherosclerotic factors (P=0.01, P<0.05, surgery+sulodexide group versus surgery+normal saline group). In addition, the levels of total cholesterol (TC), low-density lipoprotein (LDL), and free fatty acids (FFAs) were significantly downregulated by sulodexide, whereas high-density lipoprotein (HDL) levels were significantly increased. The downregulation of these atherosclerotic factors, combined with favorable changes in lipid metabolism, likely act together to reduce the occurrence of injury-related atherosclerosis, and this can be due to the effect of sulodexide on glycocalyx reconstruction.

### Sulodexide attenuates the infiltration of leukocytes to balloon-injury arteries through reconstructing glycocalyx

**Figure 6:**
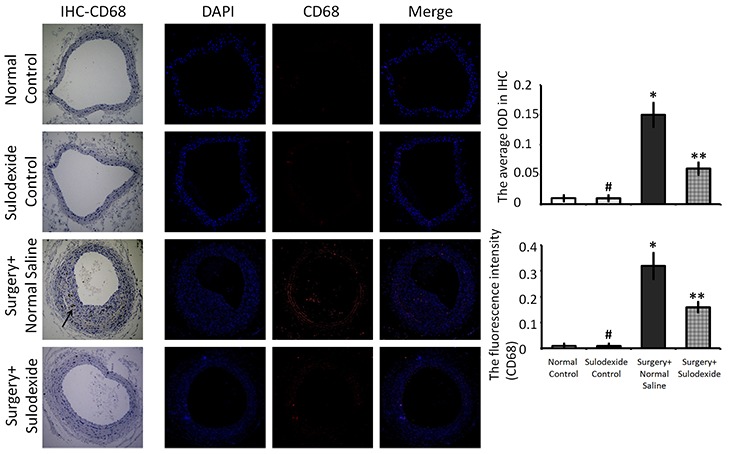
Sulodexide decreases CD68-positive inflammatory cell infiltration into blood vessels Representative IHC and IF (100 × magnification) staining for CD68. In the IHC images, the black arrow shows the infiltration of CD68-positive inflammatory cells into the blood vessel. In the IF images, the red fluorescence shows the infiltration of CD68-positive inflammatory cells into the blood vessel. The right-hand graphs show quantification of IHC and IF images. #P>0.05 sulodexide control versus normal control, *P<0.05 surgery+normal saline versus normal control, **P<0.05 surgery+sulodexide versus surgery+ normal saline.

CD68 is a marker of monocytes/macrophages and expresses in locally infiltrating inflammatory cells. Balloon-injury not only caused endothelial hyperplasia but also induced the infiltration of CD68-positive cells (P=0.001, P<0.05, surgery+normal saline group versus normal control group, Figure 6). Sulodexide attenuated the endothelial hyperplasia and further decreased the infiltration of CD68-positive cells (P=0.01, P<0.05, surgery+sulodexide group versus surgery+normal saline group). As mentioned above, an intact glycocalyx provides endothelial contact inhibition to prevent hyperplasia, and isolates endothelium from the adhesion and infiltration of inflammatory cells. We conclude that the protective effort of sulodexide against the infiltration of leukocytes is relates to its ability to aid in glycocalyx reconstructing.

## DISCUSSION

The glycocalyx forms a gel-like layer that acts as an endothelial lining and shields endothelial cells from direct contact with blood (Figure 1). Under normal physiological conditions, endothelial glycocalyx preserves the integrity of the blood vessel wall, forms contact inhibition among endothelial cells, and creates a selectively permeable structure. Glycocalyx can also serve as an active reservoir for proteins important in vascular function, including lipoprotein lipase, extracellular superoxide dismutase, anti-thrombin III and the anti-coagulant heparin sulfate [14]. This strategic position implies it has an important role in vascular function, such as anti-coagulation, anti-inflammation, endothelial proliferation, and lipid metabolism. Damage to the endothelial glycocalyx is associated with an influx of lipoproteins, increased expression of pro-inflammatory factors, and adhesion of leukocytes to the endothelium. Therefore, glycocalyx reconstruction contributes to the balance in vascular function, such as hyperplasia, coagulation, inflammatory responses, and atherosclerosis [15].

The rapid recovery in the integrity of the glycocalyx provides the protection of normal endothelial function. The thickness of normal glycocalyx has been estimated to be 0.33-0.44 mm [16]. In the electron microscopy images, it is clear that sulodexide can repaire the integrity of the glycocalyx, which had been damaged by balloon surgery (Figure 2). In contrast, in the normal saline treated surgical group, the glycocalyx had a very slow recovery. Both the damage and the delayed restoration of glycocalyx may contribute to vascular inflammation and dysfunction. Comparing the distinct organelle arrangement within the endothelial cells in the surgery+sulodexide group with the faint cytoarchitecture in the surgery+normal saline group (Figure 2), we can readily see the importance of the glycocalyx for endothelial cell function. Previous studies have also shown that blood vessel injury and pro-atherogenic stimuli had a direct impact on glycocalyx deconstruction [17, 18], which could cause endothelial dysfunction, inflammation, and dyslipidemia.

Overlying the vascular endothelium, the glycocalyx exerts a variety of functions, which are important in maintaining normal vascular physiology and inhibiting vascular diseases. With respect to the normal structure of the endothelium, the intact glycocalyx is involved in endothelial cell contact inhibition, and so prevents hyperplasia of the endothelium. As shown in Figure 3, damage to the glycocalyx, stimulates endothelial hyperplasia, which can be blocked by sulodexide, presumably by re-establishing the glycocalyx and restoring contact inhibition between endothelial cells. In normal endothelial function, the glycocalyx has an important role in transferring shear stress into shear-dependent endothelial responses, which leads to NO release [19]. The intact glycocalyx serves as the primary sensor for plasma shear stress on endothelial cells [20]. A previous study has shown that enzymatic removal of the glycocalyx resulted in reduced NO release during shear stress [21]. The NO released is synthesized by eNOS, which therefore is important in maintaining normal endothelial function, and thus plays a crucial role in regulating vascular tone, cellular proliferation, leukocyte adhesion, and platelet aggregation. In this study, despite the endothelial hyperplasia, the loss of glycocalyx reduced the expression of eNOS (Figure 3). While, sulodexide reconstructed the glycocalyx and restored the eNOS levels. These data point to the fact that in the recovery of endothelium, the increase in the number of cells is not sufficient, and that the overlying of glycocalyx plays an indispensable role in functional restoration (Figure 1). In other words, sulodexide is able to restore the endothelium both structurally and functionally, which highlights the importance of the glycocalyx in the endothelium. In summary, the intact glycocalyx reconstructed by sulodexide treatment connects the plasma environment with the endothelium, helping to regulate vascular function.

The process of coagulation relies on two important factors: platelets and fibrinogen. Platelets are derived from megakaryocytes in response to injury [22]. In our study, compared with the control group, the levels of both platelets and fibrinogen were higher in the surgery+normal saline group (Figure 3), which could be accounted for surgery-related stress and inflammatory response. In contrast, the platelet and fibrinogen levels returned to normal in the surgery+sulodexide group. This indicates the risk of thrombosis following this blood vessel injury is reduced. Platelets normally respond to damage on the vessel wall, resulting in platelet adhesion/activation and thrombosis. The biomechanical properties of glycocalyx play a prominent role in preventing blood from clotting. The restored glycocalyx therefore functions to inactivate/silence the platelets, and furthermore the gel-like property of the glycocalyx prevent the platelets from accessing the endothelium [23]. Moreover, because a narrow lumen causes changes in flow velocity and turbulence [20], the thrombus correlates with the endothelial hyperplasia that could also be due to the absence of glycocalyx. Fibrinogen plays a key role in the formation of blood clots since it acts in platelet aggregation. Acting as a scaffold, glycocalyx can simultaneously bind and activate anti-thrombin, which then inhibits the formation of blood clots by preventing the conversion of fibrinogen to fibrin [24]. In addition, heparan sulfate, which is a constituent of glycocalyx, also blocks the pro-coagulant activity of thrombin [25]. Therefore, the intact glycocalyx, which is associated with coagulation-related factors, is actively engaged in the process of blood clotting. The reduced fibrinogen seen in the surgery+sulodexide group therefore partially demonstrates the functional recovery of the repaired glycocalyx. Thus, glycocalyx repair will counteract thrombus formation following endothelial injury and its related inflammation [26].

The anti-adhesive property of glycocalyx preclude leukocytes from directly interacting with endothelial cells and so protects endothelial cells from inflammatory activation. Shedding of the glycocalyx exposes adhesive receptors on the endothelium and thus enhances inflammatory adhesion [27]. As shown in the results, sulodexide not only preserves the structural recovery of glycocalyx, but also attenuates the increased expression of the inflammatory factors CD31 and ICAM1 in the balloon-injured endothelium (Figure 4). As a member of the immunoglobulin superfamily, CD31 is a component of the intercellular junctions of activated endothelium, and is involved in leukocyte adhesion and migration [11]. ICAM1 is a cell surface glycoprotein, which typically mediates the interaction between endothelial cells and leukocytes. After balloon-injury, the damaged endothelium that lacks the glycocalyx exposes adhesive receptors and increases the contact with leukocytes. This contact then activates the endothelial cells, enhancing the expression of inflammatory factors in a positive feedback loop, which together combine to increase the adhesion and infiltration of inflammatory cells into the vascular wall. In our study, the inflammatory factors CD31 and VCAM1 were decreased by the sulodexide (Figure 4). This lowered expression of CD31 and VCAM1 cooperates with the reconstruction of glycocalyx (Figure 1 and 2), which serves to prevent direct contact between leukocytes and endothelial cells, and so attenuates the injury-induced expression of inflammatory factors in endothelium.

Damage to the glycocalyx favors the pathological adhesion and infiltration of leukocytes into the vascular wall, and during their passage through the blood wall, the leukocytes compress the endothelial glycocalyx, further damaging it and accelerating the vascular lesion. Therefore, along with endothelial healing, the reconstruction of the glycocalyx is another important factor in protection of the endothelium. As shown in this study, balloon-injury damaged glycocalyx and caused endothelial cell activation and leukocyte adhesion. Additionally, a previous study has suggested that sulodexide, in particular the glycosaminoglycan component, can bind to and in turn affect circulating leukocytes thereby down-regulating vascular inflammation [28]. As shown in Figure 4, the levels of WBCs and the inflammatory response factor CRP were elevated in the surgery+normal saline group and these increases in WBCs and CRP were attenuated by sulodexide treatment, which corporates with the effect of rebuilding glycocalyx. Overall, apart from its role in reconstructing glycocalyx, sulodexide can also exert anti-inflammatory effects, as well as effects on blood lipids, and thus it plays an important role in reducing the incidence of injury-related atherosclerosis.

There is a close relationship between atherosclerosis and the destruction of the endothelial glycocalyx. Damage to the endothelium is a precondition for atherosclerosis and the destruction of the endothelial glycocalyx plays an important role in endothelial cell dysfunction and subsequent atherosclerosis [29]. In the process of vascular disease, the levels of VCAM1 and OPN are connected with damage to the endothelial glycocalyx, and so are considered to be central to the atherosclerotic process. VCAM1 is a cell surface sialoglycoprotein that is expressed in activated endothelium where it mediates leukocyte-endothelium adhesion and signal transduction. OPN is a matricellular protein that is found in bone and binds hydroxyapatite with high affinity [30]. OPN is also expressed in other cells and tissues, such as macrophages, endothelial cells, and smooth muscle cells and plays a role in calcium deposition and pathologic calcification [12, 13]. In our previous study, OPN was involved in the vascular calcification in atherosclerosis [31]. As shown in this study, sulodexide promotes the reconstruction of glycocalyx, and thereby reduces the adhesion and infiltration of inflammatory cells to the endothelium, which subsequently inhibits the activation of endothelial cells and downregulates the expression of VCAM1 and OPN (Figure 5). The anti-atherosclerotic effect of sulodexide presumably related with glycocalyx reconstruction.

Hyperlipidemia is a risk factor for glycocalyx damage [32], as shown by the effect of oxidized LDL, which can degrade the glycocalyx structure and increase capillary permeability. Hypercholesterolemia also impairs the shear-stress response of the endothelium by damaging the glycocalyx [19]. In our study, TC, LDL and FFAs were all increased following balloon surgery and were significantly downregulated by sulodexide. In contrast, HDL levels were decreased by surgery and increased by sulodexide treatment (Figure 5D and 5E). The favorable effect of sulodexide on lipid metabolism not only reduces the occurrence of atherosclerosis, but also preserves the glycocalyx.

Inflammatory cell infiltration clearly plays an important role in the development of vascular disease. Although different tissues contain various cells of the macrophage lineage, such as monocytes, giant cells, Kupffer cells, and osteoclasts, the CD68 marker is found to be highly expressed on infiltrating macrophages [33], which can express elastase and matrix metalloproteinases, and participate in the process of atherosclerosis [34–36]. The previous study demonstrated that CD68-positive monocytes displayed increased attachment to endothelium in diabetes [37], which damaged the endothelial glycocalyx. From the immunofluorescence and immunohistochemistry analysis (Figure 6), the balloon-injured carotid artery had obvious infiltration of CD68-positive inflammatory cells and sulodexide attenuated this inflammatory infiltration due to the reconstruction of the glycocalyx. Altogether, glycocalyx not only plays a role in protecting endothelial cells from inflammatory activation and inhibiting the expression of inflammatory factors, it also acts as a molecular sieve to isolate the monocytes from infiltrating blood vessel. Moreover, as a membrane glycoprotein, CD68 can bind to LDL and participate in lipid aggregation in blood vessels, which can aggravate glycocalyx damage. Therefore, the glycocalyx treatments is important for preserving endothelial function.

The mechanism in the treatment of a dysfunctional glycocalyx by sulodexide is that sulodexide is similar to the component in glycocalyx and can provide precursor for reconstruction. The glycocalyx improvement is the precondition for microcirculatory function. As seen in the results, sulodexide treatment inhibited the neointimal hyperplasia and attenuated the expression of inflammatory factors. Some researchers have shown that sulodexide can preserve coronary vascular glycocalyx and attenuate myocardial ischemia/reperfusion injury and the deposition of CRP [38, 39]. Besides, sulodexide could mediate its reno-protective effects in contrast-induced nephropathy rats, which was accompanied with reduced oxidative stress, macrophage infiltration and apoptosis [40]. In diabetes, the glycocalyx damage has been associated with albuminuria [41]. A study reporting on the use of sulodexide in diabetic nephropathy showed that sulodexide can prevent morphologic changes in the glomeruli of diabetic rats and decrease albuminuria [42, 43]. These observations have also been supported in another study, which showed that supplementation with a mixture of heparin and dermatan sulfate, structurally similar to sulodexide, decreases proteinuria [44, 45].

In conclusion, our study demonstrates that sulodexide can improve the function of the endothelium after carotid artery balloon-injury, and decrease systemic inflammatory reactions. This protection by sulodexide is related to the reconstruction of glycocalyx, which is important for the normal physiological function of endothelial cells. All these effects suggest the potential usefulness of sulodexide in the preservation of endothelial function through glycocalyx remodeling. Additionally, the mechanism by which sulodexide promotes glycocalyx reformation and the effect of glycocalyx on vascular healing requires further research. As is known that the reconstruction of glycocalyx by sulodexide is related to the similar structure, the following research can use the structurally similar analogue of sulodexide, such as heparin and subunits, to analyze the processes of glycocalyx construction in detail. If possible, the isotope tracing methods can be used to study the action of sulodexide on glycocalyx construction.

## MATERIALS AND METHODS

### Animals and groups

Adult male Sprague-Dawley (SD) rats, aged 12 weeks, with a mean weight of 350 g, were obtained from the Laboratory Animal Center of Peking Union Medical College Hospital. All rats were housed under a standard laboratory environment at an ambient temperature of 22∼25°C, humidity of 55∼65% and a 12/12 h light/dark cycle. All rats had free access to food and water. This study complied with the Animal Management Rule of the Chinese Ministry of Health, and was approved by the Animal Care and Utilization Committee of Peking Union Medical College.

The rats were randomly divided into four groups (n=10): a normal control group with normal feeding; the sulodexide control group who received an intraperitoneal injection of sulodexide without surgery; the surgery+normal saline group who underwent carotid artery balloon-injury surgery, and were intraperitoneally injected with normal saline; and the surgery+sulodexide group who underwent carotid artery balloon-injury surgery, and were intraperitoneally injected with sulodexide. A previous report from Potter et al. found that after degradation of the glycocalyx, seven days were required for the glycocalyx to reconstruct its structure *in vivo* [46]. For this reason, rats were injected intraperitoneally with sulodexide at a dose of 2.0mg/kg for seven days.

### Carotid artery balloon-injury model

To simulate angioplasty injury in the clinic, we used a Fogarty balloon catheter (2F, Edwards Lifesciences, Irvine, CA, USA) to create the balloon-induced carotid artery injury. SD rats were anesthetized with 10% chloral hydrate (0.3mL/100g, intraperitoneal injection). The carotid artery vasculature was exposed, and an isolated arterial segment was created with hemostatic controls. The Fogarty balloon catheter was introduced into the external carotid artery through an arteriotomy incision, advanced to the common carotid artery, and then inflated to maintain two atmospheric pressure using a pressure pump (Encore 26, Boston Scientific, Natick, MA, USA), the catheter was twitched three times to damage the endothelium. During the injury process, the range of balloon scratching was limited to 2 cm. The catheter was then removed, and the incision was closed. Upon full recovery, rats were returned to the animal care facility and provided with a standard rat chow and water *ad libitum*. After one week, the rats were anesthetized by the injection of sodium pentobarbital and two centimeters of the injured carotid arteries was isolated from each animal and cleaned of surrounding tissue. Blood was also drawn to test for coagulation function, lipid metabolism, and the level of circulating leukocytes. All experimental protocols complied with guidelines of the institutional animal care and use committee.

### Electron microscopy analysis

Since the endothelial glycocalyx is a highly dynamic and fragile structure, and traditional tissue staining and perfusion-fixation procedures usually result in a loss of the glycocalyx, electron microscopy has proven to be an effective method to study its dimensions and function. During the process of collecting carotid arteries for examination by electron microscopy, glutaraldehyde fixation was used to perfuse the vascular system, which can preserve the structure of the glycocalyx. After collecting blood samples, the pre-cooled glutaraldehyde (4°C) was perfused into the aortic root. In this way, the endothelial glycocalyx in the carotid artery can be fixed and its native structure preserved. The glycocalyx thickness and integrity detected by transmission electron microscope were then used to estimate the degree of recovery of the glycocalyx.

### Hematoxylin-eosin

The harvested carotid arteries were also processed using standard procedures through a series of graded alcohols and xylene, then paraffin-embedded. Paraffin-embedded slices were serially sectioned at 4 mm intervals and mounted on slides. Parts of the carotid artery slides were stained with hematoxylin-eosin (HE), and observed by microscopy (DMI4000 B; Leica Microsystems, Wetzlar, Germany). The areas corresponding to the neointima and media were measured using Image-Pro Plus 6.0 software (Media Cybernetics, Inc., Bethesda, MD, USA), and the ratio of the intima area to media area was calculated to estimate the level of neointimal hyperplasia.

### Immunohistochemistry

Immunohistochemical (IHC) staining was performed on the sectioned carotid arteries to assess the levels of various cytokines. After blocking in phosphate-buffered saline containing 5% goat serum for 30 minutes at room temperature (20-25°C), the sections were incubated overnight at 4°C with primary antibodies (Abcam, Cambridge, England). Specific proteins were detected using the following antibodies: anti-eNOS (catalog number: ab76198), anti-osteopontin (catalog number: ab8448), anti-ICAM1 (catalog number: ab171123), anti-VCAM1 (catalog number: ab134047), anti-CD31 (catalog number: ab28364), and anti-CD68 (catalog number: ab955). The following day, signal amplification was performed with horseradish peroxidase-conjugated goat anti-rabbit IgG secondary antibody (1:500; ZhongshanJinqiao Biotechnology Co., Ltd, Beijing, China). Cytokines expression levels were quantified using integrated optical density (IOD) values generated by Image-Pro Plus 6.0 software.

### Immunofluorescence

Immunofluorescence staining was performed on the sectioned carotid arteries to assess the infiltration of inflammatory cells. Vascular tissue sections were blocked with 5% goat serum for 30 min at room temperature, then incubated with primary antibody CD68 (1:500; Abcam, Cambridge, England) overnight at 4°C. The secondary antibody used was Alexa Fluor 488 donkey anti-mouse (1:1000; Invitrogen, Life Technologies, Carlsbad, CA, USA). Sections were incubated with the secondary antibody in the dark for 2h at room temperature. The nuclei were then stained with DAPI. The localization of CD68, a monocyte/macrophage marker was assessed by fluorescence microscopy (ZEISS). Confocal images of sections were sequentially acquired with Zeiss AIM software on a Zeiss LSM 700 confocal microscope system (Carl Zeiss Jena, Oberkochen, Germany). For quantification, three randomly selected high power fields (HPFs) (400× for IHC and immunofluorescent studies) were analyzed in each section. The mean number of positively stained cells per HPF for each rat was then determined by summation of all numbers.

### Statistical analysis

All data are expressed as means ± standard error and were compared by ANOVA and Tukey post hoc test (using SPSS19.0). Differences were considered significant at P<0.05.
